# The human transmembrane mucin MUC17 responds to TNFα by increased presentation at the plasma membrane

**DOI:** 10.1042/BCJ20190180

**Published:** 2019-08-22

**Authors:** Hannah Schneider, Evelin Berger, Brendan Dolan, Beatriz Martinez-Abad, Liisa Arike, Thaher Pelaseyed, Gunnar C. Hansson

**Affiliations:** Department of Medical Biochemistry, University of Gothenburg, SE-405 30 Gothenburg, Sweden

**Keywords:** enterocyte, glycocalyx, mucin, small intestine

## Abstract

Transmembrane mucin MUC17 is an integral part of the glycocalyx as it covers the brush border membrane of small intestinal enterocytes and presents an extended *O*-glycosylated mucin domain to the intestinal lumen. Here, we identified two unknown phosphorylated serine residues, S4428 and S4492, in the cytoplasmic tail of human MUC17. We have previously demonstrated that MUC17 is anchored to the apical membrane domain via an interaction with the scaffolding protein PDZK1. S4492, localized in the C-terminal PDZ binding motif of MUC17, was mutated to generate phosphomimetic and phosphodeficient variants of MUC17. Using Caco-2 cells as a model system, we found that induction of an inflammatory state by long-term stimulation with the proinflammatory cytokine TNFα resulted in an increase of MUC17 protein levels and enhanced insertion of MUC17 and its two phospho-variants into apical membranes. Up-regulation and apical insertion of MUC17 was followed by shedding of MUC17-containing vesicles. Transmembrane mucins have previously been shown to play a role in the prevention of bacterial colonization by acting as sheddable decoys for encroaching bacteria. Overexpression and increased presentation at the plasma membrane of wild-type MUC17 and its phosphodeficient variant MUC17 S-4492A protected Caco-2 cells against adhesion of enteropathogenic *Escherichia coli*, indicating that C-terminal phosphorylation of MUC17 may play a functional role in epithelial cell protection. We propose a new function for MUC17 in inflammation, where MUC17 acts as a second line of defense by preventing attachment of bacteria to the epithelial cell glycocalyx in the small intestine.

## Introduction

The apical surface of intestinal enterocytes is covered by a dense and heavily glycosylated glycocalyx that extends up to one micrometer from the cell membrane into the lumen. Although not yet biochemically characterized, it is likely that extended transmembrane mucins MUC3, MUC12 and MUC17 are the main components. Using *in vivo* labeling of *O*-glycans we have previously shown that the murine orthologue Muc17 constitutes part of the glycocalyx in mouse small intestine [1]. The transmembrane mucins MUC3, MUC12 and MUC17 are clustered in the same genomic locus, 7q22. They contain a heavily *O*-glycosylated proline threonine serine (PTS)-rich domain that forms the extended extracellular mucin domain [2]. MUC3, MUC12 and MUC17 belong to the group of sea-urchin sperm protein, enterokinase and agrin (SEA) type transmembrane mucins due to their extracellular SEA domain between the mucin domain and the single-span transmembrane domain, which is followed by a cytoplasmic tail (CT) domain [3, 4]. The SEA domain is known to be autocatalytically cleaved during protein folding in the endoplasmic reticulum, thus rendering a mature transmembrane mucin heterodimer which is then inserted into the apical plasma membrane [5]. The SEA domain unfolds when mechanical forces are applied to the transmembrane mucin. The exact function of the SEA domain however remains yet to be defined [6].

MUC17 is expressed in the small and large intestines, where it covers the apical membranes of enterocytes [1]. Apical localization of MUC17 is regulated by PDZ domain containing 1 (PDZK1) and is dependent on the *N*-glycosylation status of the transmembrane mucin [7, 8]. Treatment with the cholinergic agonist carbachol (CCh) induces endocytosis of MUC17 in Caco-2 cells and in mouse duodenal tissue, a process that occurs alongside mucus and bicarbonate secretion [2]. Although first cloned over 25 years ago, the function and regulation of MUC17 remain largely unknown [3, 4].

Inflammatory bowel disease (IBD) is associated with a dysfunctional mucus barrier and abnormalities in gene expression of certain secreted and transmembrane mucins, including MUC17 [9, 10]. Muc2-deficient mice suffer from a disruption of the intestinal mucus layer which allows luminal bacteria to reach the epithelial cells, resulting in the spontaneous onset of colitis [11, 12], which closely mimics ulcerative colitis (UC) in human patients [13]. Mouse models of colitis exhibit augmented healing after treatment with truncated recombinant MUC17, suggesting that MUC17 can play a protective role during chronic inflammation [14, 15]. Anti-TNFα-treatment is frequently used for treating patients suffering from UC and Crohn's disease (CD), but a potential role of TNFα outside of the context of the immune system is still not fully understood [16]. Polymorphisms in genes regulating NFκB activation and TNFα-signaling have been suggested to predict therapeutic outcomes [17]. An NFκB binding site is present in the *Muc17* promoter region and studies in mice have shown that stimulation with cytokines or growth factors can induce *Muc17* gene promoter activity [3, 18]. These findings are supported by the up-regulation of MUC17 mRNA in response to TNFα [9].

MUC1, another SEA-type transmembrane mucin, has been studied in much greater detail than MUC17 due to its potential role in the onset and progression of various cancers. MUC1 has also been shown to protect against bacterial infection [19, 20]. *Muc1*^−/−^ mice are more susceptible to infection with *Campylobacter jejuni*, whereas bovine Muc1 inhibits binding of *Escherichia coli*, *Staphylococcus aureus* and *Bacillus subtilis* to Caco-2 cells [21]. During *Helicobacter pylori* infection, MUC1 prevents bacterial adhesion to the epithelium by forming a steric barrier and acting as a sheddable decoy for bacteria [22]. MUC17 has been suggested to play a role during bacterial infections, as suppression of endogenous MUC17 in human intestinal cell lines results in susceptibility to bacterial invasion [23]. The MUC17 CT comprises multiple serine, threonine and tyrosine residues, three of which have been put forward as putative phosphorylation sites which have not been previously further investigated [4]. In contrast, phosphorylation has been extensively studied in MUC1 where phosphorylation correlates with altered cancer cell properties in pancreatic and ovarian cancer cell lines [24, 25]. MUC1 phosphorylation has also been linked to bacterial infection as it is induced by *Pseudomonas aeruginosa* [26].

Here, we have studied the protective role of MUC17 in an intestinal epithelial cell model. We show that protein levels of MUC17 are up-regulated upon stimulation with TNFα and that TNFα promotes increased insertion of MUC17 into the apical plasma membrane, which is followed by a decrease in bacterial adhesion to Caco-2 cells. We have identified two novel phosphorylation sites in the MUC17 CT, one of which may regulate the cell-protective properties of MUC17. We propose that elevated levels of MUC17 at the apical membrane protect intestinal epithelial cells against bacterial binding, thus furthering our understanding of the role of MUC17 in the intestinal tract.

## Material and methods

### Plasmids

A human duodenal cDNA library (Invitrogen) was used to amplify MUC17 cDNA holding three tandem repeats using primers F 5′-CGCGGCTCTAGACCTGTGACCACTTCTTCTCCAACC-3′ and R 5′-GCGCGGAAGCTTTTAAAATGATGTCGTCATTACCTGAGG-3′. The resulting MUC17-3TR amplicon was cloned into a pSMYFP vector using XbaI and HindIII restriction sites. Cloning of adenoviral plasmids was based on the AdEasy system [27]. MUC17-3TR and YFP were amplified by PCR from template pSMYFP-MUC17-3TR. Primers for amplification of MUC17-3TR were F 5′-CGGCGTCGACCCACCATGGAGACAGACACACTCC-3′ and R 5′-GCGCGGAAGCTTTTAAAATGATGTCGTCATTACCTGAGGC-3′. YFP was amplified using primers F 5′-CGGCGTCGACCCACCATGGAGACAGACACACTCC-3′ and R 5′-GCGCGGAAGCTTTTACTTGTACAGCTCGTCCATGCCGAGA-3′. Products were inserted into pShuttle-CMV via HindIII and SalI restriction sites. The plasmid was linearized by PmeI digestion and integrated into adenoviral backbone vector pAdEasy-1 following electroporation into *E. coli* BJ5183-AD-1 (Agilent Technologies). Phosphodeficient MUC17-3TR-A and phosphomimetic MUC17-3TR-E were generated by site-directed mutagenesis of MUC17-3TR using primers F 5′-AGGCCTCAGGTAATGACGACAGCATTTTAGAAGCTTCTAGATAAGATATCC-3′ and R 5′-GGATATCTTATCTAGAAGCTTCTAAAATGCTGTCGTCATTACCTGAGGCCT-3′ (MUC17-3TR-A), and F 5′-TTCAGAGGCCTCAGGTAATGACGACAGAGTTTTAGAAGCTTCTAGATAAGATATCCGATCC-3′ and R 5′-GGATCGGATATCTTATCTAGAAGCTTCTAAAACTCTGTCGTCATTACCTGAGGCCTCTGAA-3′ (MUC17-3TR-E). Mutated MUC17-3TR cDNAs were linearized with PmeI prior to electroporation into *E. coli* BJ5183-AD-1.

### Cell culture

Caco-2 cells (ATCC HT-37) and HEK-293 cells (ATCC CRL-1573) were cultured at 37°C and 5% CO_2_ in Iscove's Modified Dulbecco's Medium (IMDM, ThermoFisher) containing 10% (vol/vol) FCS, 50 U/ml penicillin and 50 µg/ml streptomycin.

### Generation of adenovirus

Replication deficient adenoviruses for YFP, MUC17-3TR, MUC17-3TR-A and MUC17-3TR-E overexpression were generated as described elsewhere [27, 28]. Final high-titer stocks were purified by cesium chloride gradient ultracentrifugation, mixed 1 : 1 with storage buffer (10 mM NaCl, 0.1% (w/v) BSA, 50% (w/v) glycerol, 10 mM Tris pH 8.0 in H_2_O) and stored at −20°C. For titration, 44 000 HEK-293 cells/well were seeded in 100 µl IMDM in 96-well cell culture plates and infected 24 h post-seeding with a dilution series of adenovirus stocks in 100 µl IMDM. After 10 days, lysis events were counted by microscopy. Concentrations of the adenovirus stocks in plaque-forming units (PFU) were calculated according to the following formula: PFU = 0.69 × TCID_50_/ml with TCID_50_ = virus dose that infects 50% of the cultured cells [29].

### TNFα stimulation assay

Caco-2 cells were seeded at 75 000 cells/cm^2^ on multiwell plates. Twenty-three hours post-seeding cells were treated with 3 mM EGTA in OptiMEM (ThermoFisher) for 1 h, followed by YFP, MUC17-3TR, MUC17-3TR-A or MUC17-3TR-E adenovirus transduction (2.2 × 10^4^–2.2 × 10^5^ PFU/ml) in fresh OptiMEM. After 4 h an equal amount of OptiMEM was added. Twenty-four hours post-transduction, cells were washed twice in IMDM and incubated in fresh medium with 10 ng/ml TNFα for 1 h or 24 h. Medium aliquots were centrifuged at 200×***g*** for 5 min at room temperature and supernatant stored at −20°C. The remaining medium was used for vesicle isolation. Cells were harvested in PBS by scraping, pelleted at 200×***g*** for 5 min at room temperature and stored at −80°C.

### Immunoprecipitation

Immunoprecipitation of MUC17-3TR was performed on ice. Magnetic Protein G Dynabeads (Life Technologies) were washed in PBS and coated with anti-MUC17C1 antibody in PBS for 5 h at 4°C. Unbound antibody was removed by washing 5× with 1% (v/v) Igepal CA-630 (Sigma) in PBS. Alternatively, pre-coated anti-c-Myc Agarose Beads (Sigma, A7470) were used. Cells were harvested by scraping and lysed in 50 mM Tris pH 7.5, 1 M NaCl, 10 mM MgCl_2_, cOmplete™ Mini EDTA-free Protease Inhibitor Cocktail (Roche), 5% (v/v) glycerol, 1% (v/v) Triton-X 100, 1 mM EDTA and 1 : 100 phosphatase inhibitor cocktails 2 and 3 (Sigma). Debris was removed by centrifugation at 20 000×***g*** and 4°C for 10 min and supernatant bound to anti-MUC17C1 coated protein G Dynabeads or anti-c-Myc Agarose Beads at 4°C overnight. Unbound material was removed by washing in 1% (v/v) Igepal CA-630 in PBS.

### Protein fractionation by SDS–PAGE and mass spectrometry

Bound proteins were eluted from Protein G Dynabeads or anti-c-Myc Agarose Beads in 2× Laemmli buffer with DTT, reduced by boiling at 95°C for 5 min and used for SDS–PAGE. Lanes were sliced, and each gel plug transferred into a low-binding tube (Maxymum Recovery, Axygen) for in-gel digestion. Gel plugs were cut into smaller pieces, washed in 50% acetonitrile in 50 mM ammonium bicarbonate (ABC), and reduced and alkylated using 10 mM DTT and 55 mM iodoacetamide in 50 mM ABC. After additional washes in 50% acetonitrile in 50 mM ABC, pieces were dried in a speedvac for 30 min and rehydrated in trypsin digestion solution (0.5 µg trypsin per gel slice (Promega, V5111) in 25 mM ABC). Protein digestion was performed at 37°C overnight or at 50°C for 1 h. Tryptic peptides were transferred into a new tube, and remaining peptides were harvested by washing the gel pieces with 0.2% (v/v) trifluoroacetic acid in 50% (v/v) acetonitrile. Extracted peptides were dried in a speedvac, resolved in 0.1% (v/v) formic acid and subjected to reverse-phase chromatography using C18 bonded silica (Sigma, 3 M Empore) for purification. In case of absolute quantification 500 fmol of each heavy labeled peptide (IQRPQVMTTSF oxidized and non-oxidized version, SpikeTides, JPT) were added just before clean-up. Desalted peptides were eluted by 0.1% (v/v) formic acid in 60% (v/v) acetonitrile and dried until LC–MS/MS analysis. The purified peptide samples were analyzed using a QExactive hybrid quadrupole-Orbitrap mass spectrometer coupled to an Easy nano-LC system (ThermoFisher) via an electrospray source. Injected peptides of each sample were separated on a self-made packed analytical column (150 × 0.075 mm inner diameter, C18-AQ 3 µm) using an acetonitrile gradient (from 5% to 100% at 250 nL/min in 0.1% formic acid) over 60 min. Full mass spectra were acquired from 350–1750 m/z with resolution of 70 000. The eight most intense peaks (charge state ≥ 2) were fragmented using high collision dissociation, and resulting MS/MS spectra were acquired with a resolution of 35 000 and dynamic exclusion of 30 s. In the case of absolute quantification, a separate MS method using parallel reaction monitoring (PRM) mode was used to fragment only the target ions. Reference fragmentation spectra of standard peptides (IQhRPQVMTTpSF, IQRPQVMTpTSF and IQRPQVMpTTSF, SpikeTides, JPT) were obtained by direct infusion using identical fragmentation settings as applied in the MS method used for sample analysis.

### Protein identification and quantification

MS spectra were matched against the human UniProt database (version May 2015), or an in-house database (http://www.medkem.gu.se/mucinbiology/databases/) containing the sequences of MUC17-3TR variants including human Uniprot sequences. Raw files were loaded into MaxQuant (version 2.1.4.2), files originating from one lane during SDS–PAGE were set as fractions, and carbamidomethyl was set as fixed, and oxidation of methionine as well as phosphorylation of serine, threonine, and tyrosine chosen as variable modification. Trypsin/P was selected as an enzyme. Skyline version 4.1 was used for quantification of the ratio between light and heavy peptide IQRPQVMTTSF.

### Antibodies

MUC17-3TR was detected using an anti-MUC17C1 polyclonal antibody raised in rabbit against C-terminal peptide CSLRHIDPETKIRIQRPQVMTTSF or a monoclonal mouse anti-Myc antibody from 9E10.2 hybridoma cells (CRL-1729, ATCC). For immunoprecipitation, anti-MUC17C1 polyclonal antibody was used. Actin was detected with a primary mouse-anti-Actin C4 antibody (Millipore, MAB1501R). Bacteria were stained with goat anti-Lipid A (ThermoFisher, PA1-73178). Secondary antibodies for immunostaining and FACS were from ThermoFisher: goat anti-mouse Alexa Fluor 488 (A-11029), donkey anti-goat Alexa Fluor 488 (A-11055), donkey anti-mouse Alexa Fluor 647 (A-31571). Horseradish peroxidase (HRP) secondary antibodies for immunoblotting were from Southern Biotech: goat-anti-mouse HRP (1034-05), goat anti-rabbit HRP (4030-05).

### SDS–PAGE and immunoblotting

Samples were boiled in 2× Laemmli buffer with DTT and proteins separated using a 10% polyacrylamide gel. Transfer to PVDF membranes (Millipore) was done by semi-dry blotting and confirmed by Ponceau-Red staining (0.1% (w/v) Ponceau-S, 1% (v/v) acetic acid). Blocking and antibody incubation was carried out in 5% milk in PBS-T (0.1% (v/v) Tween-20 in PBS). Primary antibodies were incubated at 4°C overnight: rabbit anti-MUC17C1 antibody 1 : 2000, mouse anti-Myc antibody 1 : 50, mouse anti-actin-C4 antibody 1 : 20 000. Secondary antibodies were incubated for 1–2 h at room temperature: anti-rabbit-HRP antibody 1 : 10 000, anti-mouse-HRP antibody 1 : 5 000. Blots were developed using enhanced Immobilon Western chemiluminescent HRP substrate (Millipore) and imaged with a LAS 4000 analyzer (Fujifilm). Relative quantification was performed by densitometry using ImageJ v1.49 (https://imagej.nih.gov/ij/).

### Immunofluorescence staining and microscopy

Caco-2 cells cultured on glass cover slips were washed with PBS, fixed in 4% (w/v) paraformaldehyde in PBS for 10–20 min at room temperature and transferred to a humidity chamber. Samples were either first permeabilized in 0.1% (v/v) Triton-X 100 in PBS for 10 min, followed by blocking in 5% (v/v) FCS in PBS for 1 h at room temperature or directly transferred to blocking solution for staining of extracellular material. Primary and secondary antibodies were diluted in blocking solution. Primary antibody incubation was done at 4°C overnight: mouse anti-myc 1 : 10, goat anti-Lipid A 1 : 50. Secondary antibody incubation was done for 1 h at room temperature: goat anti-mouse Alexa Fluor 488 1 : 1000, donkey anti-goat Alexa Fluor 488 1 : 1000, donkey anti-mouse Alexa Fluor 647 1 : 1000. Cell nuclei were counterstained with 2 µg/ml Hoechst-34580 (Sigma) and samples were mounted in Prolong Gold antifade (ThermoFisher). Images were acquired with a Zeiss LSM700 upright confocal microscope (Carl Zeiss) and analyzed using Imaris software v7.6.3 (Bitplane).

### Flow cytometric analysis

Caco-2 cells were harvested in Accutase® solution (Sigma) and washed in PBS. For surface expression analysis, cells were incubated with mouse anti-Myc antibody 1 : 100 for 30 min at 4°C, washed twice with FACS buffer (2% (w/v) FCS, 5 mM EDTA, 25 mM HEPES in Hank's Balanced Salt Solution (HBSS)) and labeled with goat anti-mouse IgG Alexa Fluor 488 1 : 500 for 30 min at 4°C. Cells were washed twice with FACS buffer and analyzed with a FACS JAZZ cytometer (BD). To analyze total expression, Caco-2 cells were fixed after the surface staining in 2% paraformaldehyde (Sigma) for 10 min at room temperature, washed twice with PBS and permeabilized with 0.1% saponin (Sigma) in PBS (permeabilization buffer) for 5 min. Antibody incubation was done as above, using permeabilization buffer for washing and FACS buffer for analysis. Dead cells were excluded from analysis using Fixable Viability Dye eFluor® 780 (ThermoFisher). Endogenous levels of c-Myc were analyzed in Caco-2 cells stimulated with virus storage buffer as control.

### Quantitative real-time PCR

Caco-2 cells were stimulated with TNFα for 24 h as described above. Total RNA was isolated using the RNeasy Plus Mini Kit (Qiagen) and reverse transcribed to cDNA with the High-Capacity cDNA Reverse Transcription Kit (Applied Biosystems). Quantitative real-time PCR was performed on a CFX96 Real-Time PCR Detection System (Bio-Rad) using SsoFast^TM^ EvaGreen® Supermix (Bio-Rad). Primer sequences for detection of endogenous *MUC17* were F 5′-TCTCAGCACGTTAGGACAGGT-3′ and R 5′-TCGAGGTCATCTCAGGGTTGG-3′ and for *MUC17-3TR* and its phospho-variants F 5′-CTGATCAGCGAGGAGGACC-3′ and R 5′-GGAGTTGTTGAAAGGGTGCT-3′. *YFP* transcripts were detected using primers F 5′-CACATGAAGCAGCACGACTT-3′ and R 5′-GTCTTGTAGTTGCCGTCGTC-3′. Transcripts were normalized to *RPL32* using primers F 5′-ATGCCCAACATTGGTTATGG-3′ and R 5′-CTCTTTCCACGATGGCTTTG-3′ and to *SDHA* detected with primers F 5′-TGGGAACAAGAGGGCATCTG-3′ and R 5′-CCACCACTGCATCAAATTCATG-3′. Three independent experiments were performed with each sample measured in triplicates. The analysis was performed according to the $2^{-\Delta \Delta C_{T}}$ method [30].

### Vesicle isolation

Vesicles were isolated from the freshly harvested cell culture medium by centrifugation at 4°C. Shed cells were pelleted at 2000×***g*** for 25 min, the supernatant transferred to a new tube and cell debris pelleted at 10 000×***g*** for 30 min. Vesicle isolation was done by ultracentrifugation at 100 000×***g*** for 70 min. Vesicles were washed in PBS at 100 000×***g*** for 70 min, boiled in 2× Laemmli buffer with DTT [31] and stored at −20°C.

### Cytotoxicity assay

Caco-2 cells were seeded on 24-well plates at 75 000 cells/cm^2^, transduced with MUC17-3TR, MUC17-3TR-A or MUC17-3TR-E adenovirus and treated with 10 ng/ml TNFα for 1 h or 24 h. Treatment with 5% (v/v) Triton-X 100 served as negative control. AlamarBlue® solution was added at 1 : 10 to the medium and incubated at 37°C for 30 min. Fluorescence was measured at 545/590 nm using a CLARIOstar plate reader (BMG Labtech).

### Bacterial culture

Enteropathogenic *E. coli* (CCUG 38068; EPEC) was routinely cultured on LB agar at 37°C. For use in binding assays, EPEC were grown in LB broth at 37°C.

### Bacterial adherence assay

Caco-2 cells expressing MUC17-3TR plasmids were cultured as described previously. EPEC were grown to mid-log phase in liquid culture and collected by centrifugation. Bacteria were resuspended in IMDM containing 10% (v/v) FBS to OD_600 _= 0.25. Caco-2 cultures were washed gently in sterile PBS followed by incubation with 500 µl of bacterial suspension (∼1 × 10^8^ cfu) for 1 h at 37°C and 5% CO_2_. Following incubation, monolayers were washed 3 times with sterile PBS and fixed with 2% (w/v) paraformaldehyde for 10 min at room temperature. For quantification of bacterial binding images of infected Caco-2 cells were segmented into MUC17-3TR-positive and -negative areas using the isosurface mapping function of Imaris software v7.6.3 (Bitplane). The number of bacteria adhering to MUC17-3TR-positive and -negative cells was then quantified using Imaris. Adherence assays were performed on at least three separate occasions.

### Statistics

Data are presented as mean ± standard error of the mean. One-way ANOVA or Student's *T*-test was applied using GraphPad Prism v7.02 (La Jolla, CA). Values of *P *≤ 0.05 were considered significant.

## Results

### The MUC17 CT comprises two phosphorylation sites

**Figure 1.**
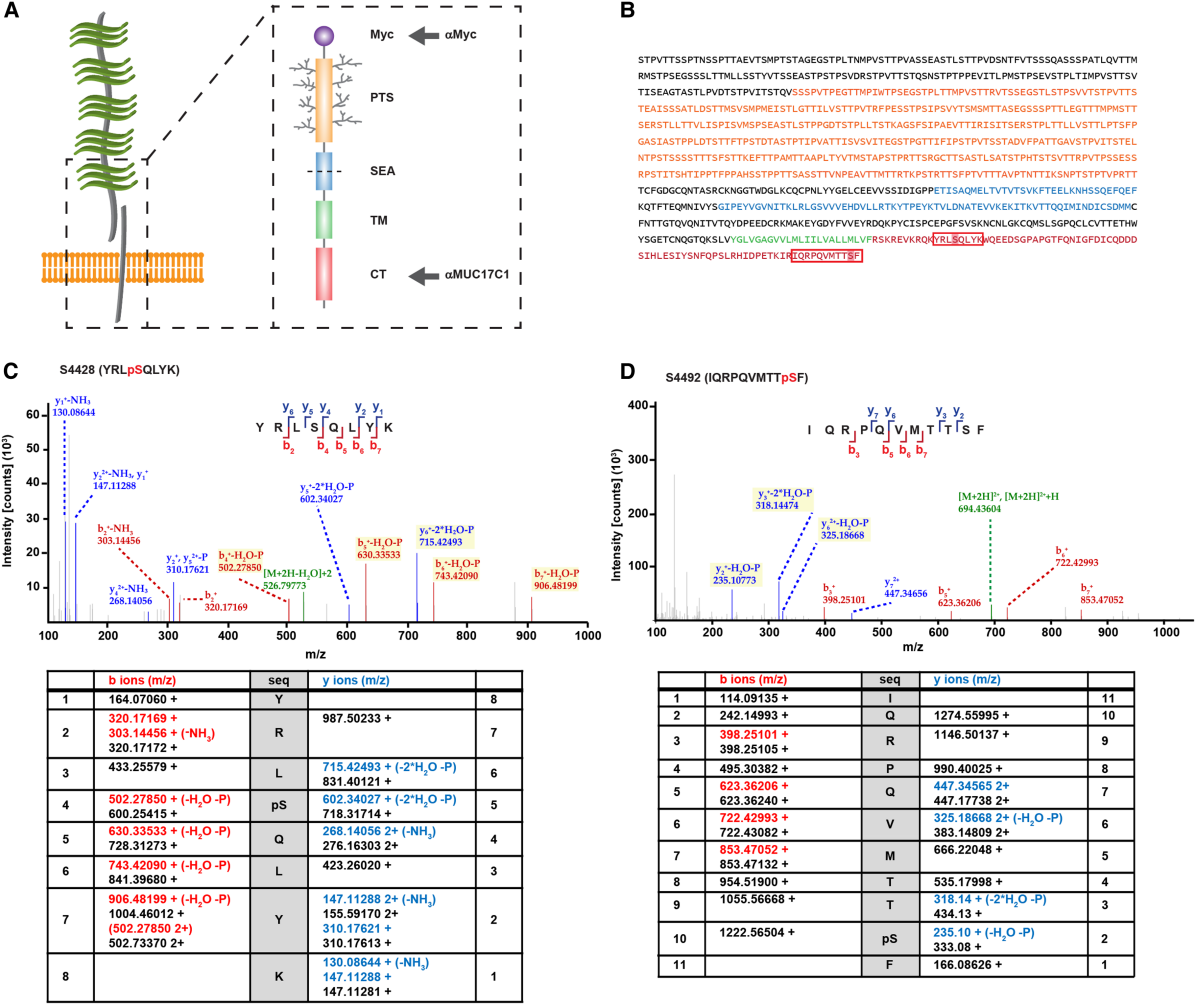
Human MUC17 comprises two phosphorylation sites in its C-terminal domain. (**A**) A recombinant MUC17 plasmid, MUC17-3TR, containing a N-terminal Myc-tag, a PTS domain of three tandem repeats (3TR), a SEA domain, followed by a transmembrane domain (TM) and a CT was expressed in Caco-2 cells. Arrows indicate epitopes recognized by antibodies. (**B**) Full-length sequence of MUC17-3TR with domains highlighted in orange (PTS), blue (SEA), green (TM) and red (CT). Amino acid sequences with the identified phosphorylation sites are framed in red boxes. (**C** and **D**) MUC17-3TR was overexpressed in Caco-2 cells and purified by immunoprecipitation. Samples were subjected to SDS–PAGE, bands cut out, trypsinized, and analyzed by mass spectrometry. Two peptides with one phosphorylation site each, pS4428 and pS4492, were identified within the CT of MUC17-3TR. Representative MS/MS spectra of detected fragment ions of MUC17 peptides YRLpSQLYK and IQRPQVMTTpSF are shown. Fragment ions potentially carrying a phosphate group are marked in yellow, precursor peptides are highlighted in green. Tables below the spectra display identified fragments of the b/y ion series in red/blue together with the non-modified (theoretical) mass to charge values in black.

To study the role of MUC17 in the intestinal epithelium, we designed a recombinant human MUC17 plasmid, termed MUC17-3TR, comprising a Myc-tagged truncated PTS domain with 3 out of 60 tandem repeats, followed by the full-length SEA domain, transmembrane domain and the CT (Figure 1A,B). We used an adenoviral expression system [27] to introduce MUC17-3TR into Caco-2 cells, a human intestinal enterocyte-like cell line routinely utilized to investigate epithelial cell barrier function [32–34].

**Figure 2.**
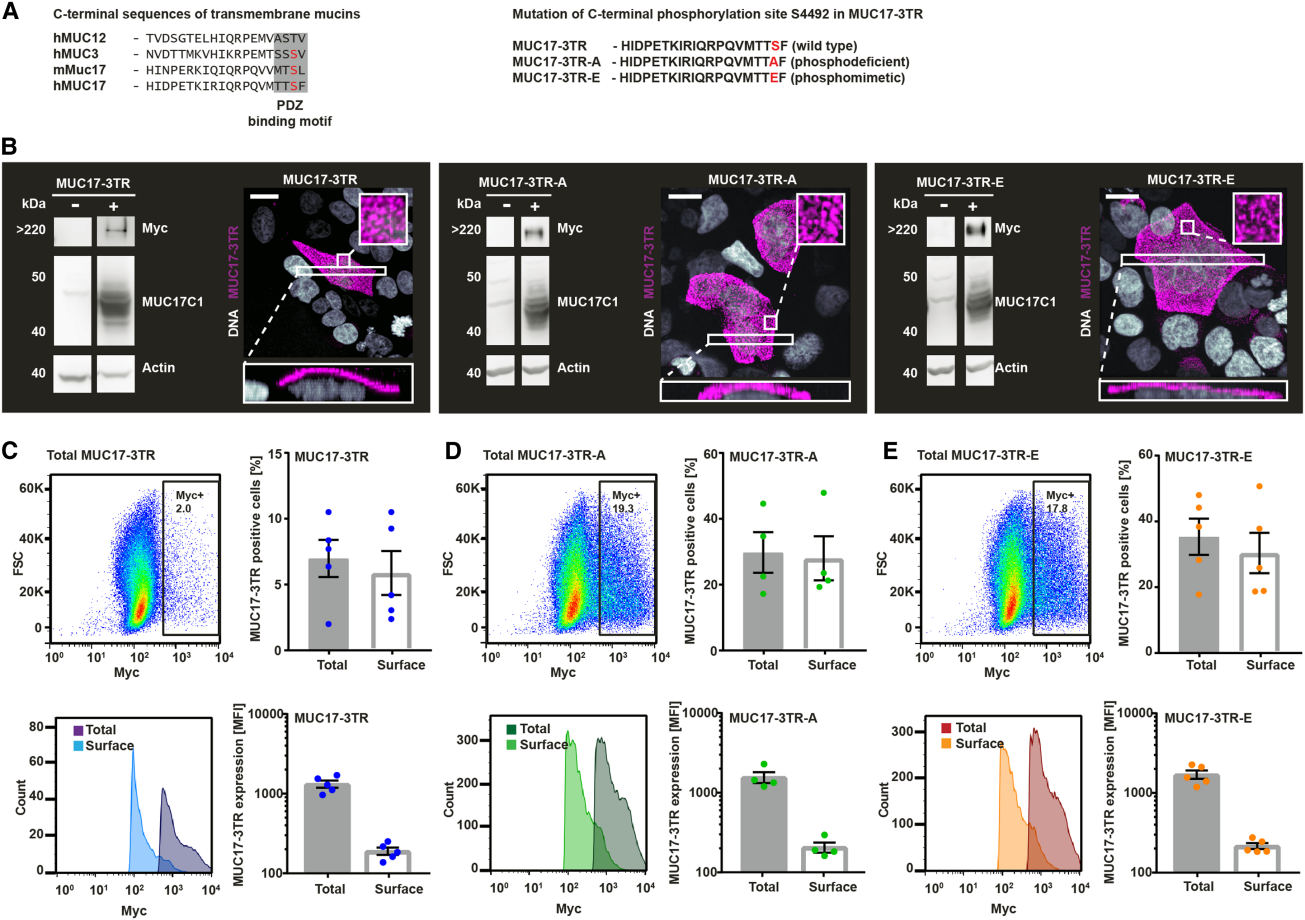
A phosphodeficient and a phosphomimetic variant of MUC17-3TR are correctly expressed and localize to the plasma membrane of Caco-2 cells. (**A**) Alignment of C-terminal sequences of MUC17 with human transmembrane mucins MUC3 and MUC12 and mouse orthologue Muc17. Phosphorylation site pS4492 lies within the PDZ binding motif. S4492 of MUC17-3TR was mutated to alanine (A) or glutamic acid (E) to generate a phosphodeficient (MUC17-3TR-A) and a phosphomimetic (MUC17-3TR-E) variant. (**B**) Whole-cell lysates from Caco-2 cells expressing MUC17-3TR, MUC17-3TR-A and MUC17-3TR-E were analyzed by immunoblot. Protein expression was evaluated using an anti-Myc mAb targeting the extracellular N-terminal Myc-tag (>220 kDa corresponding to the Myc-PTS-SEA fragment) and an anti-MUC17C1 pAb detecting the intracellular C-terminal domain (40 kDa corresponding to the SEA-TM-CT fragment). Actin was used as a loading control. Unpermeabilized Caco-2 cells overexpressing MUC17-3TR, MUC17-3TR-A and MUC17-3TR-E were stained for MUC17-3TR (anti-Myc mAb, magenta) and DNA (Hoechst, gray) and analyzed by confocal microscopy. Orthogonal projections of boxed in areas are depicted below, magnifications of boxed in areas are shown as insets. Scale bars, 20 µm. (**C**–**E**) Caco-2 cells expressing MUC17-3TR (**C**), MUC17-3TR-A (**D**) and MUC17-3TR-E (**E**) were subjected to FACS analysis. Surface expression of MUC17-3TR and its phospho-variants was detected in unpermeabilized Caco-2 with anti-Myc mAb. Following permeabilization, total MUC17 expression was analyzed using the same antibody. Total and surface expression of MUC17-3TR and its phospho-variants was observed in a comparable percentage of cells. Dot plots and histograms of representative experiments are shown. (*n *= 4–5).

The cytoplasmic part of MUC17 contains potential phosphorylation sites and we have previously demonstrated that its localization to the plasma membrane is stabilized through a direct interaction between a C-terminal PDZ binding motif and the scaffold protein PDZK1 [7]. We immunoprecipitated MUC17-3TR from Caco-2 cell lysates and analyzed the CT for potential phosphorylation sites using mass spectrometry. Two peptides, YRLSQLYK and IQRPQVMTTSF, were identified as containing one phosphorylated serine each at positions 4428 and 4492, respectively (Figure 1C,D and Supplementary Figure S1). Phosphorylation site S4492 was localized at position −1 within the C-terminal PDZ binding motif of MUC17 and was conserved in human transmembrane mucin MUC3 as well as in the MUC17 mouse orthologue Muc17 (Figure 2A).

To study the role of S4492 phosphorylation on MUC17, we generated two plasmids encoding a phosphomimetic glutamate (MUC17-3TR-E) or a phosphodeficient alanine (MUC17-3TR-A) substitution of S4492 (Figure 2A). Immunoblot analysis under reducing conditions yielded two protein fragments in all three plasmid constructs as a result of the cleaved SEA-domain: a large N-terminal fragment of >220 kDa detected by the Myc-tag and a C-terminal fragment of 45 kDa detected by an anti-MUC17C1 antibody raised against an epitope in the CT. No signal for endogenous MUC17 was detected in un-transduced Caco-2 cells, indicating indiscernible levels of endogenous MUC17 expression (Figure 2B). Confocal microscopy of MUC17-3TR and its phosphomimetic and phosphodeficient variant (phospho-variants) confirmed that all three proteins were correctly processed and targeted to the plasma membrane of Caco-2 cells (Figure 2B).

To characterize MUC17-3TR expression levels and localization in greater detail, Caco-2 cells overexpressing MUC17-3TR, MUC17-3TR-A and MUC17-3TR-E were subjected to FACS analysis. Quantification of total and surface MUC17-3TR resulted in equivalent numbers of cells, indicating that cells which express MUC17-3TR or either of the phospho-variants can also target the transmembrane mucin to the plasma membrane (Figure 2C–E). MFI for total MUC17-3TR was higher than for surface-localized MUC17-3TR, demonstrating that only some of the total amount of MUC17 protein is inserted into the plasma membrane at a given time. Comparable results were obtained for phosphomimetic MUC17-3TR-A and phosphodeficient MUC17-3TR-E (Figure 2C–E). Background signal from endogenous MYC in control Caco-2 cells was negligible (Supplementary Figure S2).

### TNFα stimulation up-regulates MUC17 expression

**Figure 3.**
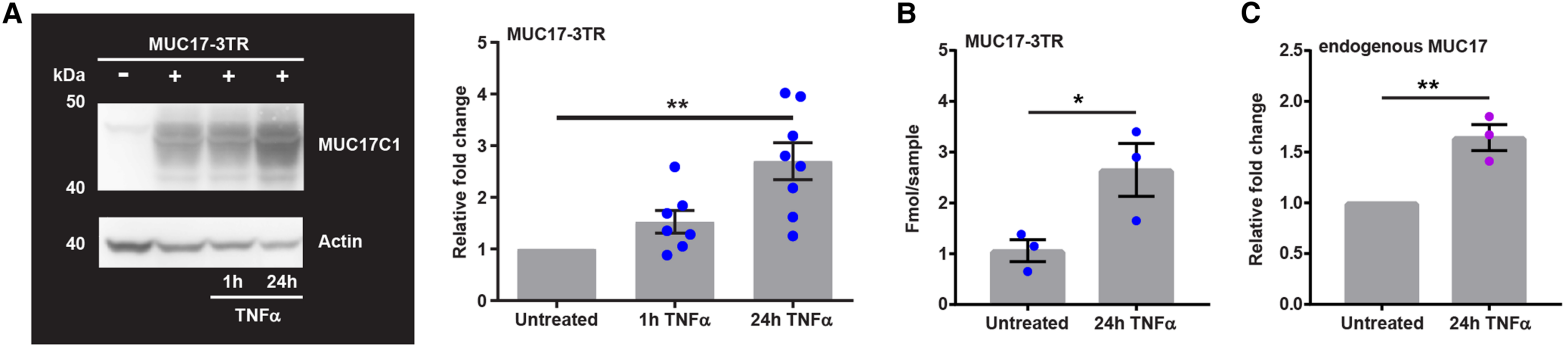
Human MUC17-3TR protein levels are increased in response to TNFα-stimulation. (**A**) Caco-2 cells expressing MUC17-3TR were stimulated with TNFα and whole-cell lysates subjected to immunoblot analysis. MUC17-3TR was detected using an anti-MUC17C1 pAb against the intracellular C-terminal domain (40 kDa: SEA-TM-CT fragment) and actin was used as a loading control. MUC17-3TR protein amounts were significantly increased following 24 h TNFα-incubation as quantified by densitometric analysis. (*n *= 7–8; **P ≤* *0.01) (**B**) Caco-2 cells were transduced with MUC17-3TR adenovirus and stimulated with TNFα for 24 h. Whole-cell lysates were subjected to SDS–PAGE, bands cut out, trypsinized, and analyzed by mass spectrometry using standard peptides for MUC17 quantification. TNFα-treatment resulted in increased amounts of MUC17-3TR in Caco-2 cells. (*n *= 3; *P ≤* *0.05) (**C**) Transcripts of endogenous MUC17 in Caco-2 cells were analyzed by quantitative real-time PCR. Following 24 h TNFα-treatment, transcript levels of endogenous MUC17 were significantly increased compared with unstimulated controls. (*n *= 3; **P ≤ 0.01).

It has previously been shown that stimulation with TNFα up-regulates MUC17 mRNA expression, thus suggesting a link to CD and UC [9]. We decided to assess short-term (1 h) and long-term (24 h) effects of TNFα-treatment on MUC17-3TR in Caco-2 cells. MUC17-3TR protein levels were increased after stimulation with TNFα for 24 h but remained unaffected by short-term TNFα-treatment (Figure 3A). Actin and total protein amounts in Caco-2 cells were unchanged by MUC17-3TR overexpression and TNFα-incubation (Supplementary Figure S3). Absolute protein quantification using mass spectrometry confirmed a 3-fold increase in MUC17-3TR protein levels after 24 h of TNFα-stimulation compared with untreated MUC17-3TR control cells (Figure 3B). Quantitative RT-PCR revealed up-regulation of MUC17-3TR mRNA and its phospho-variants MUC17-3TR-A and MUC17-3TR-E in response to 24 h TNFα-stimulation (Supplementary Figure S4A). Importantly, this effect was not mediated by the CMV promotor upstream of MUC17-3TR, as a YFP reporter under control of the same promoter was not significantly changed by 24 h stimulation with TNFα (Supplementary Figure S4B). Additionally, quantitative real-time PCR analysis demonstrated a significant up-regulation of endogenous MUC17 in Caco-2 following 24 h TNFα-treatment (Figure 3C). We concluded that long-term stimulation with TNFα up-regulates MUC17-3TR protein levels.

### C-terminal phosphorylation of MUC17 does not modulate its response to TNFα-stimulation

**Figure 4.**
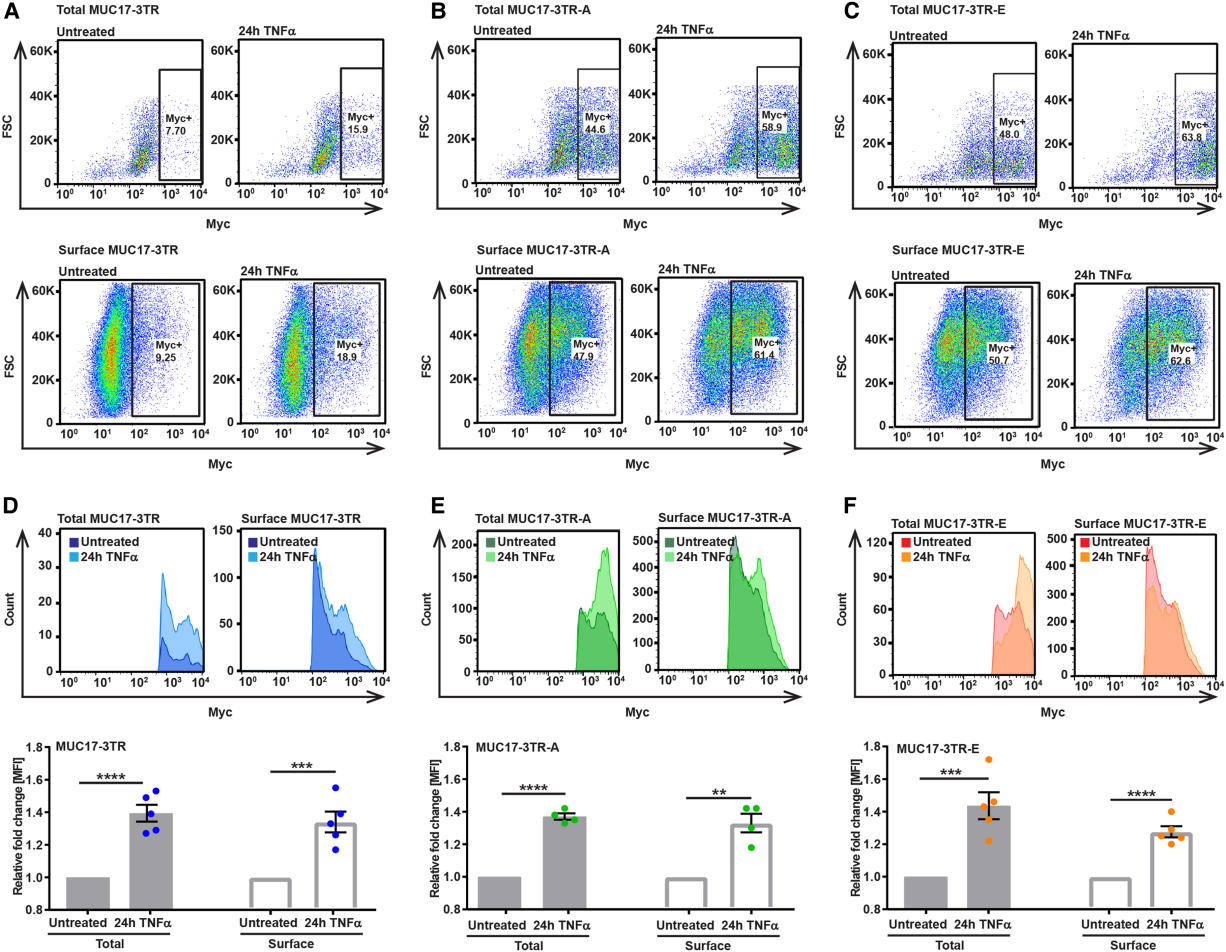
Twenty-four hours TNFα-stimulation increases protein levels of MUC17-3TR at the cell surface of Caco-2 cells. Caco-2 expressing MUC17-3TR, MUC17-3TR-A and MUC17-3TR-E were stimulated with TNFα for 24 h and subjected to FACS analysis. Surface expression of MUC17-3TR and its phospho-variants was detected in unpermeabilized Caco-2 using anti-Myc mAb and compared with total MUC17-3TR expression in permeabilized cells using the same antibody. (**A**–**C)** Changes in cell populations with total and surface MUC17-3TR expression in untreated controls and following 24 h TNFα-incubation. Expression of surface and total protein amounts of MUC17-3TR, MUC17-3TR-A and MUC17-3TR-E was observed in a higher number of Caco-2 cells following stimulation. Dot plots of a representative experiment are shown. (**D**–**F**) Relative fold change in mean fluorescence intensity (MFI) values for Caco-2 cells expressing MUC17-3TR, MUC17-3TR-A and MUC17-3TR-E without stimulation and after 24 h TNFα-treatment. Following incubation with TNFα, intensities of total and cell surface MUC17-3TR, MUC17-3TR-A and MUC17-3TR-E proteins were significantly increased. Values for MUC17-3TR were calculated comparing the TNFα-stimulated samples to unstimulated controls. Histograms of a representative experiment are shown. (*n *= 4–5; **P ≤ 0.01, ***P ≤ 0.001 or ****P ≤ 0.0001).

Next, we investigated whether the effect of TNFα-stimulation on MUC17 protein levels was affected by phosphorylation in the MUC17-3TR CT. As recombinant MUC17-3TR likely exists as both, phosphorylated and non-phosphorylated species, we assessed the effect of treatment with TNFα on each individual phospho-variant separately. Caco-2 cells overexpressing MUC17-3TR, MUC17-3TR-A or MUC17-3TR-E were stimulated for 24 h with TNFα and subjected to FACS analysis. Following TNFα-treatment quantities of surface and total MUC17-3TR expressing Caco-2 were significantly increased. A similar effect was observed for phosphodeficient MUC17-3TR-A and phosphomimetic MUC17-3TR-E (Figure 4A–C and Supplementary Figure S5). MFI for total as well as surface-localized MUC17-3TR and its phospho-variants was likewise up-regulated after stimulation with TNFα (Figure 4D–F), indicating that C-terminal phosphorylation does not modulate the response of MUC17-3TR to TNFα-stimulation.

Next, we investigated the presence of MUC17-3TR in cell culture medium as a result of maintaining plasma membrane homeostasis during MUC17-3TR overexpression. MUC17-3TR was detected in the spent culture medium from Caco-2 cultures following long-term TNFα-stimulation (Supplementary Figure S6A). Measurements of cell viability ruled out cell death as a possible cause for the presence of MUC17 in the culture medium (Supplementary Figure S7). Earlier studies have suggested that transmembrane mucins can be shed from cells while tethered to extracellular vesicles [35, 36]. Consequently, we hypothesized that a fraction of plasma membrane-localized MUC17-3TR is shed from Caco-2 cells when attached to vesicles. Vesicle preparations of spent culture supernatants using ultracentrifugation showed increased levels of MUC17-3TR, independent of the phosphorylation of S4492, in the culture medium after 24 h TNFα-stimulation (Supplementary Figure S6B,C). The presence of MUC17-3TR-A/E in the culture medium was not the result of cell damage as cell viability was unaffected (Supplementary Figure S7).

### Membrane-localized MUC17-3TR prevents bacterial adhesion to Caco-2 cells

**Figure 5.**
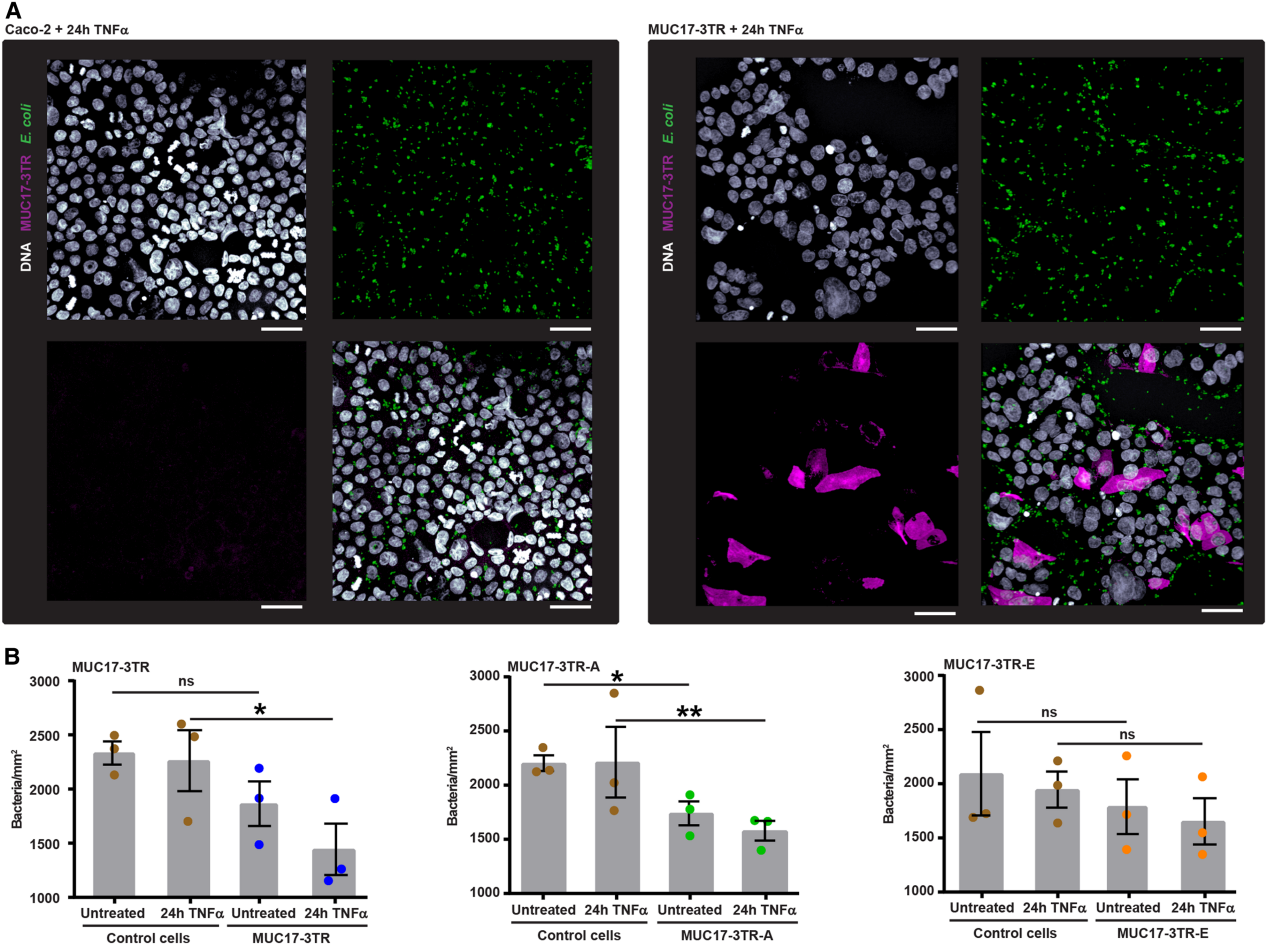
Increased presentation of MUC17-3TR at the cell surface prevents bacterial binding to Caco-2 cells. (**A**) Control and MUC17-3TR expressing Caco-2 cells were stimulated with TNFα for 24 h, incubated with EPEC for 1 h, permeabilized and stained for MUC17-3TR (anti-Myc mAb, magenta), *E. coli* (anti-Lipid A, green) and DNA (Hoechst, gray). Samples were analyzed with confocal microscopy to calculate the number of bacteria adhering to MUC17-3TR-positive and negative cells. Representative images are shown. Scale bars, 50 µm. (**B**) Quantification of *E. coli* bound to the surface of Caco-2 cells expressing MUC17-3TR, MUC17-3TR-A and MUC17-3TR-E in untreated controls and after 24 h TNFα-stimulation. Bacterial binding was significantly reduced in MUC17-3TR-A overexpressing cells under control conditions and in MUC17-3TR and MUC17-3TR-A overexpressing cells following TNFα-treatment. (*n *= 3 biological replicates shown, *n *= 3 technical replicates per biological replicate; *P ≤ 0.05 or **P ≤ 0.01).

MUC17 has been suggested to play a role in cell restitution [14] and protection of epithelial cells from *E. coli* infection [23]. We hypothesized that increased insertion of MUC17-3TR into the cell membrane in response to TNFα protects cell surfaces against bacterial attachment. To test this hypothesis, we designed an immunofluorescence based assay to measure binding of EPEC to Caco-2 cells expressing recombinant MUC17-3TR in comparison with control cells. In order to exclude any influence from endogenous MUC17, we took advantage of the Myc-tag to exclusively detect MUC17-3TR. Following 24 h TNFα-stimulation, we observed a significant reduction in the number of EPEC on the surface of MUC17-3TR-positive Caco-2 compared with non-expressing control cells (Figure 5A).

For quantification of bound bacteria, we infected Caco-2 with MUC17-3TR, MUC17-3TR-A and MUC17-3TR-E and stimulated cells with TNFα for 24 h followed by incubation for 1 h with EPEC. After correction for unspecific binding of bacteria to glass coverslips (Supplementary Figure S8), we observed a significant reduction in bacterial binding to Caco-2 cells overexpressing MUC17-3TR-A and a trend towards decreased binding in Caco-2 cells overexpressing MUC17-3TR. Stimulation with TNFα further diminished bacterial binding to Caco-2 cells expressing MUC17-3TR and MUC17-3TR-A (Figure 5B).

Together, these results suggest a relationship between TNFα-induced MUC17-3TR levels on the plasma membrane and MUC17-3TR-mediated protection against the bacterial binding.

## Discussion

The intestinal epithelium is constantly challenged by luminal bacteria. While the significance of the secreted mucus layer for protection against bacteria has been widely recognized, the function of the glycocalyx covering the intestinal epithelium remains less well understood [12, 37, 38]. The transmembrane mucin MUC17 together with MUC3, MUC12 and MUC13 constitutes an integral component of the epithelial cell glycocalyx. Previous studies have linked altered transmembrane mucin expression to IBD and have suggested protective functions for MUC17 during intestinal inflammation [23, 39, 40]. Here, we have studied the role of MUC17 in a TNFα-induced inflammatory state in Caco-2 cells and demonstrate that MUC17 protects intestinal epithelial cells against bacterial attachment.

First, we overexpressed a recombinant MUC17-3TR transmembrane mucin with a truncated mucin domain and verified its correct processing and targeting to the plasma membrane. Using mass spectrometry, we identified two novel phosphorylation sites, S4428 and S4492, in the MUC17-3TR CT. One of these, S4492, lies within the PDZ binding motif. We have previously shown that MUC17 is stabilized in the enterocyte apical membrane through interaction with PDZK1 [7] and phosphorylation of PDZ-binding motifs is known to weaken binding to PDZ proteins [41, 42]. We, therefore, hypothesized that the phosphorylation status of S4492 regulates direct interaction between MUC17-3TR and PDZ proteins, thus at least in part controlling membrane localization of MUC17. However, mutation of S4492 to a phosphomimetic glutamate or a phosphodeficient alanine did not affect the presentation of MUC17-3TR at the plasma membrane of Caco-2 cells, suggesting that the specific interaction between MUC17 and PDZK1 is not governed by phosphorylation at S4492 to an extent at which membrane localization of MUC17 is affected.

TNFα is a key player in inflammatory processes and a major target in IBD therapy. Our study demonstrated that TNFα caused elevated intracellular and surface-localized MUC17-3TR protein levels in Caco-2 cells following 24 h of stimulation. MUC17-3TR mRNA levels were likewise increased after 24 h of TNFα-treatment, pointing to TNFα-dependent regulation of MUC17 on both transcriptional and post-transcriptional levels. Our results are in accordance with a previous study, showing the up-regulation of endogenous MUC17 in LS174T cells after 24 h TNFα-treatment [9]. Reports also suggest that the expression of transmembrane mucin MUC1 is up-regulated in response to stimulation with interleukins and TNFα [43–45], proposing a functional link between inflammation and up-regulation of transmembrane mucins.

Previous studies have shown that phosphorylation of transmembrane mucin MUC1 plays a role in inflammation and bacterial infection and could be stimulated by *P. aeruginosa* [26]. Moreover, MUC1 phosphorylation promotes interaction between MUC1 and TLR5, which result in inhibition of downstream TLR5 signaling [46]. Our FACS analysis of MUC17-3TR and the two phospho-variants of S4492 revealed that all three proteins responded similarly to TNFα-treatment, demonstrating that phosphorylation at S4492 does not control the sensitivity of MUC17 to TNFα-stimulation. As the function of the second identified phosphorylation site S4428 remains undefined, it is conceivable that the co-ordinated phosphorylation of S4492 and S4428 might be required to regulate the MUC17-3TR response to TNFα-signaling.

In small intestinal enterocytes, MUC17 is localized to the tip of the microvilli at the upper region of intestinal villi [2]. In healthy individuals, intestinal bacteria are kept at a safe distance from the enterocytes through a combination of efficient peristalsis and transport of luminal content towards distal regions, mucus secretion by goblet cells and secretion of antibacterial peptides that safeguard intestinal crypts. Disruption of this equilibrium results in commensal and pathogenic bacteria coming in close contact with the intestinal epithelium. Pathogens, in particular, have evolved methods to circumvent host protective mechanisms. Under disease conditions, increased density of surface glycocalyx transmembrane mucins could contribute to guarding the apical epithelial surface through steric hindrance whereas a decreased density of transmembrane mucins might facilitate bacterial attachment and subsequent invasion. Down-regulation of MUC17 by siRNA in cell lines with endogenous MUC17 expression resulted in increased invasion by enteroinvasive *E. coli* (EIEC) [23]. Mice infected with *Trichuris muris* show increased expression of the Muc17, Muc4 and Muc13 mucins which contribute to enhance the intestinal glycocalyx [47]. To test whether augmented levels of MUC17-3TR affected bacterial binding, we challenged Caco-2 cells with EPEC and quantified bound bacteria. Overexpression of MUC17-3TR reduced bacterial attachment, suggesting that MUC17 might have a cell protective effect.

Interaction between mucins and bacteria has been addressed in multiple studies [48, 49], revealing two opposing mechanisms; binding and shielding. Binding of bacteria is dependent on the specificities of bacterial adhesins towards glycans presented by transmembrane mucins. Adhesins with specificity for host carbohydrate epitopes can mediate bacterial binding and promote infection and invasion. On the other hand, the highly hydrated carbohydrate-rich glycocalyx increase the separation of bacteria and the epithelial cell membrane and thus shield cells from bacterial attachment. The latter scenario suggests a protective function for transmembrane mucins as has been observed in studies of MUC1 in the stomach [22, 50]. We propose a similar function for MUC17 in the small intestine, as a result of increased presentation at the plasma membrane in response to TNFα. Transmembrane mucins reach furthest out from the epithelial cell into the intestinal lumen and will be the most likely first binding targets for bacteria with specificity for host glycoproteins. Once bacteria attach to the transmembrane mucin, the host requires a mechanism for removing the bacteria. Vesicle shedding from the apical part of microvilli is believed to be especially important and frequent in the small intestine, suggesting that the enterocytic microvillus is a ‘vesicle-generating organelle’ [51]. Vesicles with a single membrane harboring the extracellular part of transmembrane mucins are shed into the intestinal lumen. Indeed, the presence of transmembrane mucins MUC1, MUC4 and MUC16 has been confirmed in vesicles secreted by human tracheobronchial epithelia and has been suggested to play a role in innate mucosal defenses [52]. This system is severely perturbed in *Myo1α* knock-out mice, suggesting a coupling between the generation of vesicles and microvilli [51]. Recently a mechanistic link between the glycocalyx and membrane shape regulation was suggested by an increased production of vesicles in HeLa-cells with high endogenous Muc1 expression [53].

A study of MUC1 in breast cancer cells suggested the existence of MUC1-containing sub-populations of lipid rafts which contribute to its secretion via exosomes [54]. Lipid rafts are well-known signaling platforms and have proven to play a critical role in TNFα-dependent signaling pathways [55]. The idea of linking the secretion of transmembrane mucins in vesicles to lipid rafts during inflammation is striking and would open a whole new perspective on potential signaling pathways. The increased amounts of MUC17 in the form of vesicles shed from the TNFα-treated cells suggest that TNFα could indeed enhance this process. However, it is also likely that the process of shedding MUC17 in vesicles represents a protective mechanism of the cell in order to avoid crowding of overexpressed membrane protein in the plasma membrane. Which of these possibilities applies remains open for further studies.

**Figure 6.**
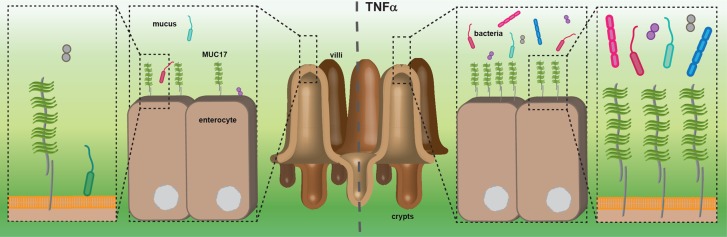
Proposed model for the protective role of MUC17 in the small intestine. The transmembrane mucin MUC17 is expressed on small intestinal enterocytes where it constitutes part of the glycocalyx. The overlying loose and penetrable mucus layer is mainly composed of the secreted mucin MUC2 and harbors commensal bacteria. During inflammation, increased numbers of bacteria are present in the functionally impaired mucus layer and in closer contact with epithelial cells. MUC17 protein expression is augmented in response to TNFα-signaling, resulting in an increased presentation at the plasma membrane. MUC17 limits bacterial access to the cell membrane and prevents bacterial adherence to epithelial cells by steric hindrance.

In summary, our study demonstrates that MUC17 membrane expression protects intestinal epithelial cells from bacterial binding, which is enhanced by TNFα-signaling. We suggest that MUC17 acts as a second line of defense if bacteria breach the mucus layer in the small intestine. During inflammation caused by a dysfunctional mucus layer more bacteria are penetrating down to the epithelium [11, 56]. We propose that increased levels of MUC17 help protect the intestinal epithelium from bacterial binding by steric hindrance (Figure 6). As host-microbe interactions are complex, the relationship between the proinflammatory role of TNFα and the beneficial effect of anti-TNFα-treatment in IBD remains the topic of future studies.

## Abbreviations

ABC: ammonium bicarbonate
CCh: carbachol
CD: Crohn's disease
CT: cytoplasmic tail
EIEC: enteroinvasive *E. coli*
EPEC: enteropathogenic *E. coli*
HRP: Horseradish peroxidase
IBD: inflammatory bowel disease
PDZK1: PDZ domain containing 1
PFU: plaque forming units
PRM: parallel reaction monitoring
PTS domain: proline threonine serine-rich domain
PTS: proline threonine serine
SEA domain: sea-urchin sperm protein, enterokinase and agrin domain
UC: ulcerative colitis
