# Supplementary material for: The human transmembrane mucin MUC17 responds to TNFα by increased presentation at the plasma membrane
Source: Biochem J. 2019 Aug 22;476(16):2281–95. doi: 10.1042/BCJ20190180 (PMC6705488; doi:10.1042/BCJ20190180)
Supplement: Supplementary Figures [file BCJ-476-2281-s1.pdf]

SUPPLEMENTAL TO

Figure S1

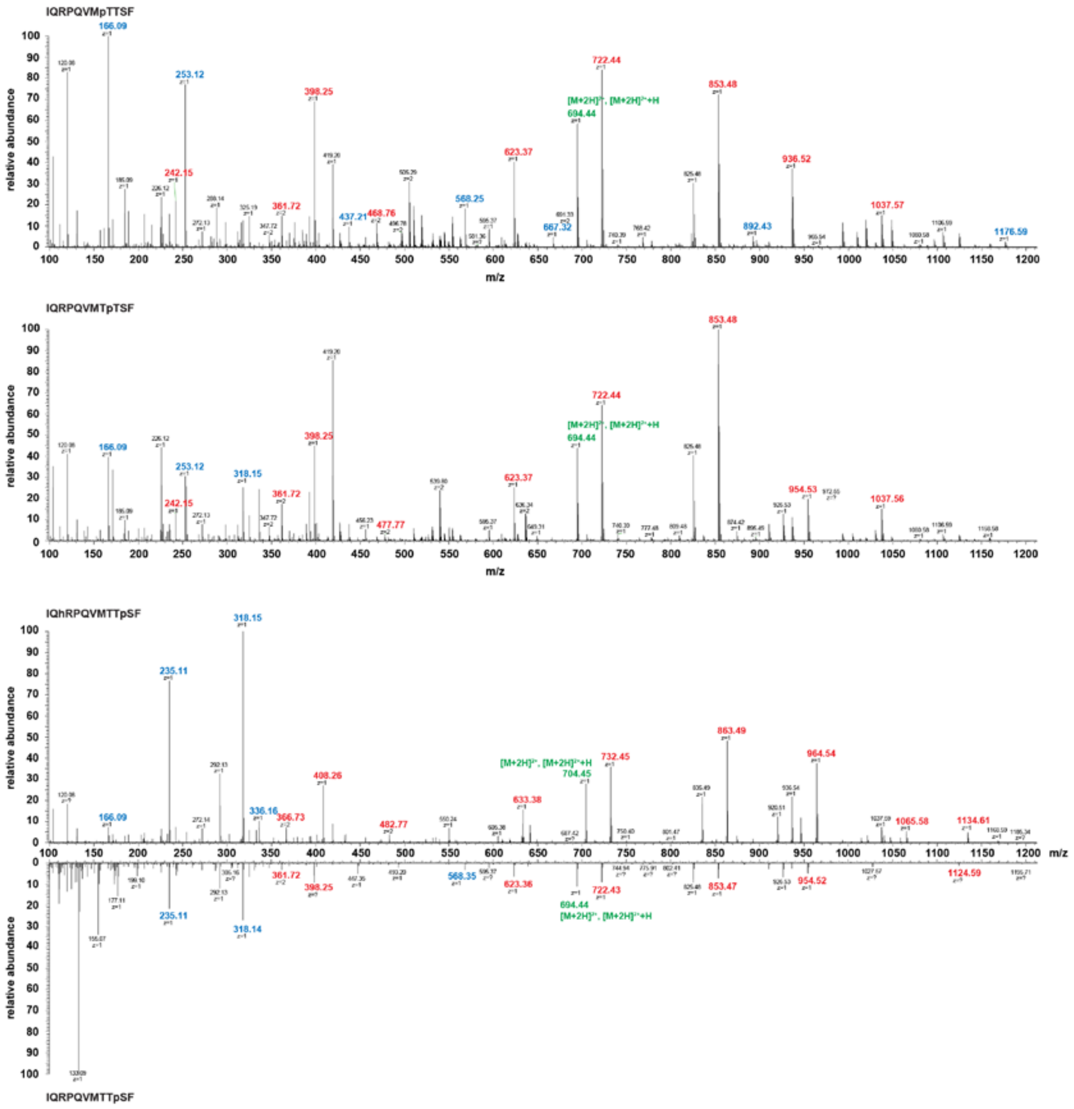

IQRPQVMpTTSF

	b-H₃PO₄	b ions (m/z)		y ions (m/z)	y-H₃PO₄	
1	---	---	I	---	---	11
2	---	242.15 +	Q	1274.56 + 637.78 2+	1176.58 + 588.80 2+	10
3	---	398.25 + 199.63 2+	R	1146.50 + 573.75 2+	1048.52 + 524.77 2+	9
4	---	495.30 + 248.16 2+	P	990.40 +	892.42 +	8
5	---	623.36 + 312.18 2+	Q	893.35 +	795.37 +	7
6	---	722.43 + 361.72 2+	V	765.29 +	667.31 +	6
7	---	853.47 + 427.24 2+	M	666.22 +	568.24 +	5
8	936.51 + 468.76 2+	1034.49 + 517.75 2+	pT	535.18 +	437.20 +	4
9	1037.56 + 519.28 2+	1135.53 + 568.27 2+	T	354.17 +	---	3
10	1124.59 + 562.80 2+	1222.57 + 611.79 2+	S	253.12 +	---	2
11	---	---	F	166.09 +	---	1

IQRPQVMTpTSF

	b-H₃PO₄	b ions (m/z)		y ions (m/z)	y-H₃PO₄	
1	---	---	I	---	---	11
2	---	242.15 +	Q	1274.56 + 637.78 2+	1176.58 + 588.80 2+	10
3	---	398.25 + 199.63 2+	R	1146.50 + 573.75 2+	1048.52 + 524.77 2+	9
4	---	495.30 + 248.16 2+	P	990.40 +	892.42 +	8
5	---	623.36 + 312.18 2+	Q	893.35 +	795.37 +	7
6	---	722.43 + 361.72 2+	V	765.29 +	667.31 +	6
7	---	853.47 + 427.24 2+	M	666.22 +	568.24 +	5
8	---	954.52 + 477.76 2+	T	535.18 +	437.20 +	4
9	1037.56 + 519.28 2+	1135.53 + 568.27 2+	pT	434.13 +	336.16 + 318.14 + (-H₂O)	3
10	1124.59 + 562.80 2+	1222.57 + 611.79 2+	S	253.12 +	---	2
11	---	---	F	166.09 +	---	1

IQhRPQVMTTpSF

	b-H₃PO₄	b ions (m/z)		y ions (m/z)	y-H₃PO₄	
1	---	---	I	---	---	11
2	---	242.15 +	Q	1284.56 + 642.78 2+	1186.58 + 593.80 2+	10
3	---	408.25 + 204.63 2+	hR	1156.50 + 578.75 2+	1058.52 + 529.77 2+	9
4	---	505.30 + 253.16 2+	P	990.40 +	892.42 +	8
5	---	633.36 + 317.18 2+	Q	893.35 +	795.37 +	7
6	---	732.43 + 366.72 2+	V	765.29 +	667.31 +	6
7	---	863.47 + 432.24 2+	M	666.2205	568.24 +	5
8	---	964.52 + 482.76 2+	T	535.18 +	437.20 +	4
9	---	1065.57 + 533.29 2+	T	434.13 +	336.16 + 318.14 + (-H₂O)	3
10	1134.59 + 567.80 2+	1232.57 + 616.79 2+	pS	333.08 +	235.11 +	2
11	---	---	F	166.09 +	---	1

Fig. S1. Identification of the MUC17 C-terminal phosphorylation site S4492 within peptide IQRPQVMTTSF.

Caco-2 cells overexpressing MUC17-3TR were harvested and subjected to immunoprecipitation, followed by SDS-PAGE, trypsinization of bands and mass spectrometry analysis. MS/MS spectra of the sample and standard peptides (average

of 50 subsequent fragmentation spectra) for all three potential phosphorylation sites (T4490, T4491, S4492) are presented. A mirror plot of MS/MS spectra comparing the MUC17-3TR sample with standard peptide IQhRPQVMTTpSF demonstrates phosphorylation at S4492. Tables show identified fragments for all standard peptides with major identified fragments of the b/y ion series labeled in red/blue and theoretical mass to charge values in black.

Figure S2

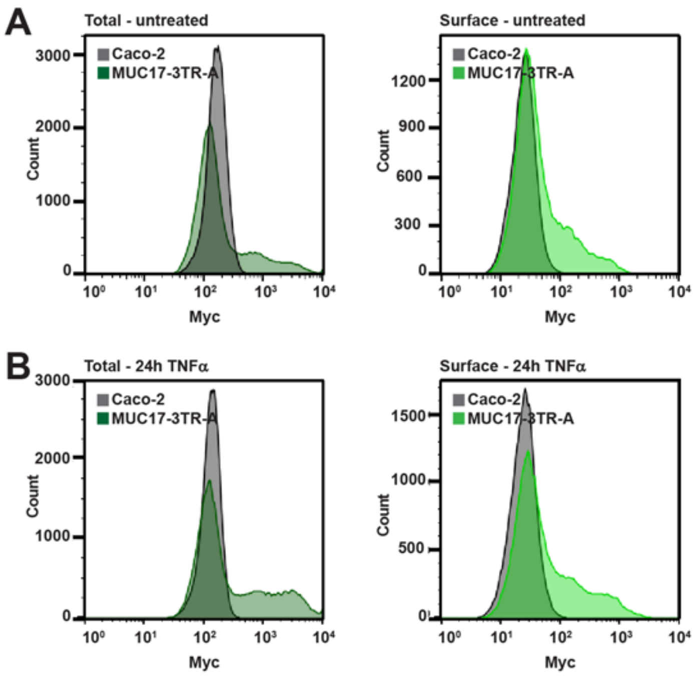

Fig. S2. Surface and total MUC17-3TR expressing Caco-2 can be clearly distinguished from control cells.

MUC17-3TR-A was overexpressed in Caco-2. Cells were left untreated **(A)** or stimulated with TNFα for 24 h **(B)** followed by FACS analysis using anti-Myc mAb. Surface localized MUC17-3TR was detected in unpermeabilized cells and total MUC17-3TR was analyzed following permeabilization. MUC17-3TR overexpressing Caco-2 showed a distinct signal for MYC-positive cells under all tested conditions. Histograms of a representative experiment are shown.

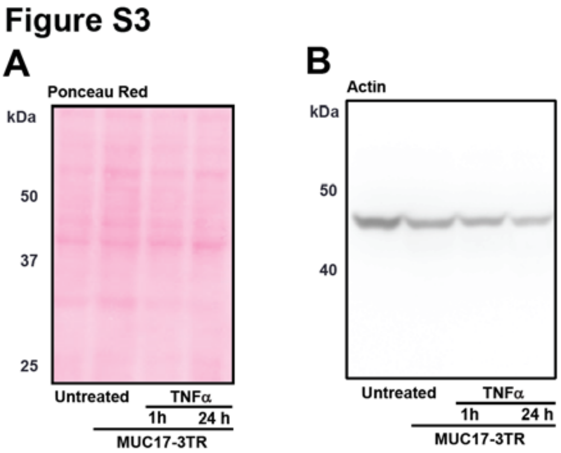

Fig. S3. Overexpression of MUC17-3TR in Caco-2 cells has no significant effect on actin and total protein amounts.

Caco-2 cells were transduced with MUC17-3TR adenovirus and stimulated with TNFα for 1 h or 24 h. Untreated Caco-2 cells served as controls. Whole cell lysates were analyzed by immunoblot. **A)** Ponceau Red staining confirmed equal protein amounts in all samples. **B)** Actin was detected using an anti-actin-C4 antibody. A small decrease was observed in adenovirus transduced cells in comparison to control cells.

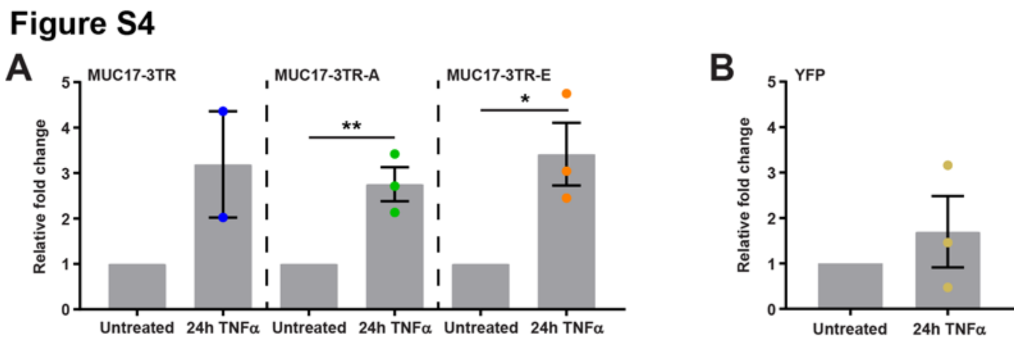

Fig. S4. MUC17-3TR mRNA quantities are increased following 24 h TNFα-treatment.

A) MUC17-3TR, MUC17-3TR-A and MUC17-3TR-E were overexpressed in Caco-2 and cells stimulated with TNFα for 24 h. Transcripts of MUC17 were subjected to quantitative real-time PCR analysis. Expression of MUC17-3TR-A and MUC17-3TR-E was significantly augmented following TNFα-stimulation. MUC17-3TR demonstrated a trended increase. (*n*=2-3; *$P\leq 0.05$ or **$P\leq 0.01$) **B)** YFP adenovirus was used in a control experiment to transduce Caco-2 cells, followed by TNFα-treatment for 24 h. Quantitative real-time PCR analysis showed no significant change of YFP mRNA quantities in stimulated samples when compared to controls. (*n*=3)

Figure S5

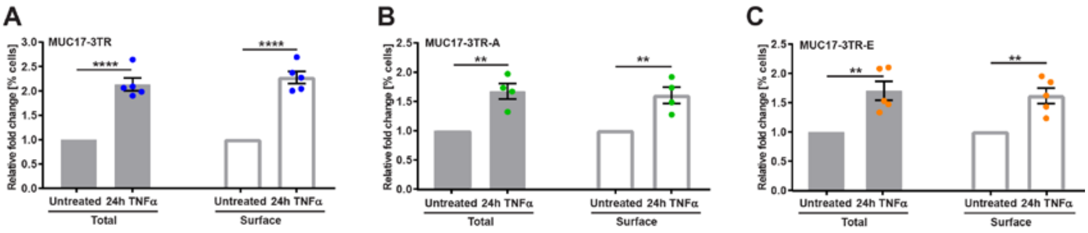

Fig. S5. 24 h TNFα-stimulation increases the number of surface and total MUC17-3TR positive cells.

Caco-2 cells overexpressing MUC17-3TR **(A)**, MUC17-3TR-A **(B)** or MUC17-3TR-E **(C)** were stimulated with TNFα for 24 h and compared to untreated controls using FACS analysis. Surface localized MUC17-3TR was detected in unpermeabilized Caco-2 using anti-Myc mAb and total MUC17-3TR expression was quantified in permeabilized cells using the same antibody. Following TNFα treatment, an increased number of cells expressing total and surface localized MUC17-3TR was determined.

Figure S6

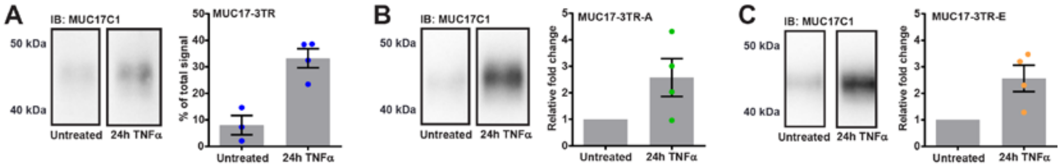

Fig. S6. MUC17-3TR is detected in spent cell culture medium following 24 h TNFα-stimulation.

A) Elevated levels of MUC17-3TR protein were detected in cell culture medium following incubation with TNFα for 24 h. MUC17-3TR in cell culture medium from Caco-2 was analyzed by immunoblot with anti-MUC17C1 pAb. Non-stimulated Caco-2 cells expressing MUC17-3TR served as control. Values represent the proportion of the sample signal compared to the total signal of all samples in an individual experiment. (n=3-4) **B, C)** Protein amounts of MUC17-3TR-A and MUC17-3TR-E in vesicles isolated from Caco-2 medium were increased following 24 h TNFα-stimulation. Caco-2 cells overexpressing MUC17-3TR-A and MUC17-3TR-E were treated with TNFα for 24 h and spent cell culture medium was harvested. Vesicles were isolated from the spent cell culture medium by ultracentrifugation and samples analyzed by gel electrophoresis and immunoblot using anti-MUC17C1 pAb. (*n*=4)

Figure S7

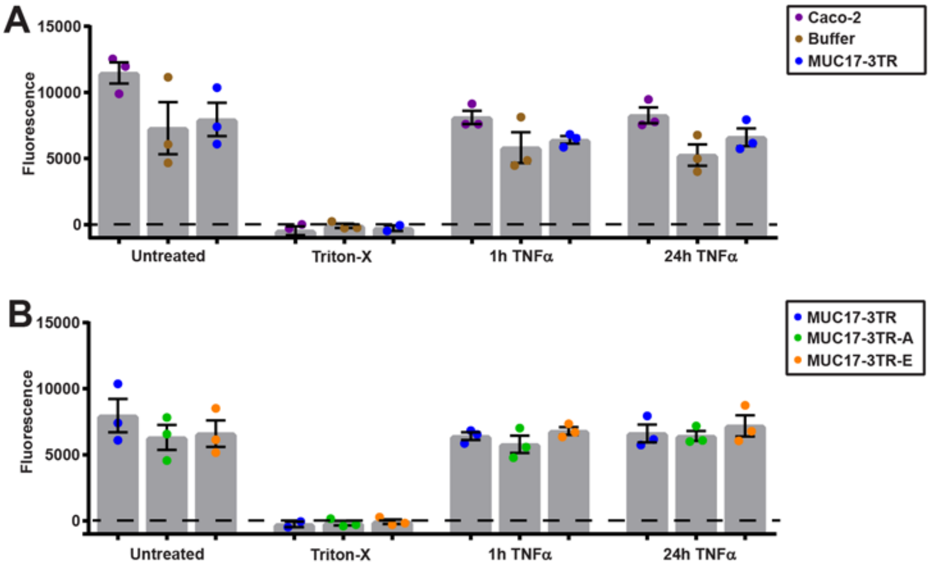

Fig. S7. Caco-2 cell viability is not impaired by transduction with MUC17-3TR adenovirus or TNFα-treatment.

Caco-2 cells were transduced with MUC17-3TR, MUC17-3TR-A or MUC17-3TR-E adenovirus and stimulated with TNFα. Alamar Blue® solution was added to the cell culture medium to analyze changes in metabolic activity using fluorescence measurements. Triton-X treated cells served as negative control. **A)** Caco-2 cells transduced with MUC17-3TR adenovirus or mock-treated with adenovirus storage buffer showed a small decrease in metabolic activity compared to controls. Treatment with TNFα had no significant effect on cell viability in comparison to untreated controls. (*n*=3) **B)** No differences in metabolic activity could be detected in Caco-2 cells overexpressing MUC17-3TR, MUC17-3TR-A or MUC17-3TR-E. Stimulation with TNFα had likewise no effect on cell viability. (*n*=3)

Figure S8

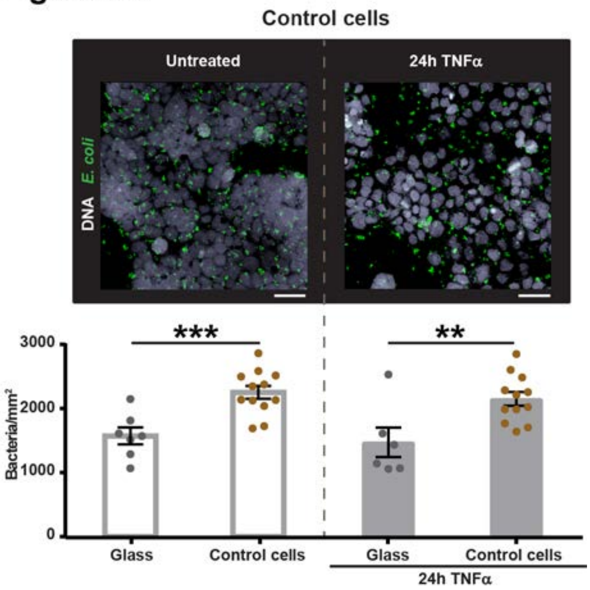

Fig. S8. Enteropathogenic *E. coli* specifically bind to Caco-2 cells.

Caco-2 cells were left untreated or stimulated with TNFα for 24 h, incubated with EPEC for 1 h and stained for *E. coli* (green) and DNA (gray). Samples were analyzed by confocal microscopy to quantify the number of bacteria adhering to glass cover slips and Caco-2 cells. Significantly more bacteria bound to the surface of Caco-2 cells than to glass. Scale bars, 50 µm. (n=6-12, cells: 3 technical replicates per data point, glass: 1-3 technical replicates per data point, $^{**}P \leq 0.01$ or $^{***}P \leq 0.001$)
